# *Linc-RA1* inhibits autophagy and promotes radioresistance by preventing H2Bub1/USP44 combination in glioma cells

**DOI:** 10.1038/s41419-020-02977-x

**Published:** 2020-09-15

**Authors:** Jieling Zheng, Baiyao Wang, Rong Zheng, Jian Zhang, Chunyue Huang, Ronghui Zheng, Zhong Huang, Wenze Qiu, Mengzhong Liu, Kaijun Yang, Zixu Mao, Aimin Ji, Yawei Yuan

**Affiliations:** 1 Department of Radiation Oncology, Affiliated Cancer Hospital & Institute of Guangzhou Medical University, Guangzhou, Guangdong Province People’s Republic of China; 2 Department of Radiation Oncology, Fujian Medical University Union Hospital, Fuzhou, Fujian Province People’s Republic of China; 3 Department of Radiation Oncology, Sun Yat-Sen University Cancer Center, Guangzhou, Guangdong Province People’s Republic of China; 4 Department of Neurosurgery, Nanfang Hospital, Southern Medical University, Guangzhou, Guangdong Province People’s Republic of China; 5 Department of Pharmacology and Chemical Biology, School of Medicine, Emory University, Atlanta, USA

**Keywords:** CNS cancer, CNS cancer, Long non-coding RNAs, Long non-coding RNAs

## Abstract

Radiotherapy is one of the standard treatments for glioma patients; however, its clinical efficacy is limited by radioresistance. We identified a mechanism of such resistance mediated by *linc-RA1* (radioresistance-associated long intergenic noncoding RNA 1). *Linc-RA1* was upregulated in radioresistant glioma cells and glioma tissue samples, compared with radiosensitive cells and nontumor tissues. *Linc-RA1* was associated with inferior overall survival and advanced clinical stage of glioma*. Linc-RA1* promoted glioma radioresistance in vitro and in vivo. Mechanistically, *linc-RA1* stabilized the level of H2B K120 monoubiquitination (H2Bub1) by combining with H2B and inhibiting the interaction between H2Bub1 and ubiquitin-specific protease 44 (USP44), which inhibited autophagy, thus contributing to glioma radioresistance. These results reveal that *linc-RA1*-mediated autophagy is a key mechanism of radioresistance and is an actionable target for improving radiotherapy efficacy in patients with glioma.

## Introduction

Gliomas are the most common primary brain tumors and are classified as grades I–IV under the World Health Organization (WHO) grading system^1^. High-grade glioma (HGG), including WHO grades III and IV, is the most fatal brain tumor in adults, and its treatment has been largely unsatisfactory^2^. Radiotherapy is one of the limited treatment options with verified clinical efficacy for patients with HGG^3^. Unfortunately, the efficacy of radiotherapy for HGG patients is at best modest, due to radioresistance of the tumor, the underlying mechanisms of which remain poorly characterized^4^.

Long noncoding RNAs (lncRNAs) are noncoding transcripts containing more than 200 nucleotides that can control the expression of a gene at the transcriptional, post-transcriptional, or epigenetic levels^5^. Increasing evidence has shown that specific lncRNAs are implicated in the onset and progression of various cancers^6^. For example, dysregulated lncRNAs, including lncRNA *HULC* and *CAMTA1*, can be closely linked to key aspects of pathology, progression, and outcomes in liver cancer^7,8^. Specific lncRNAs, including lncRNA *PCAT-1* and *MALAT1*, are critically involved in the development and drug resistance in gastric cancer^9,10^. Importantly, lncRNAs are considered as critical players in the tumorigenesis and progression of gliomas^11^. For example, lncRNA *HOTAIR*, with a higher expression level in glioma tissues than in nontumor tissues, is essential for glioma proliferation^12^. LncRNA *CRNDE* can promote the growth and invasion of glioma cells through the mammalian target of rapamycin (mTOR) signaling pathway^13^. However, the roles of lncRNAs in radioresistance of glioma remain largely unknown^14^. In our previous study, we used two human glioma cell lines M059J and M059K, which were derived from the same patient, with M059K cells being more resistant to irradiation (IR) than M059J cells^15^. The differential expression profile of lncRNAs between M059J and M059K cells was analyzed using an lncRNA microarray. We proved that lncRNA *SNHG18* promoted radioresistance of glioma cells by suppressing semaphorin5A^16^.

In the present study, we continued to explore the other differentially expressed lncRNAs, which are considered to be involved with the radioresistance to glioma. We identified an lncRNA and named it radioresistance-associated long intergenic noncoding RNA 1 (*linc-RA1*). The results demonstrated that *linc-RA1* is highly expressed in radioresistant glioma cells and glioma tissues, compared with radiosensitive cells and nontumor tissues, and promotes radioresistance of glioma. Mechanistically, *linc-RA1* could stabilize the level of H2B K120 monoubiquitination (H2Bub1), thereby inhibiting the activation of autophagy and contributing to the radioresistance of glioma cells.

## Results

### *Linc-RA1* is upregulated in glioma radioresistant cell lines and correlates with advanced glioma grades and poor prognosis

**Fig. 1:**
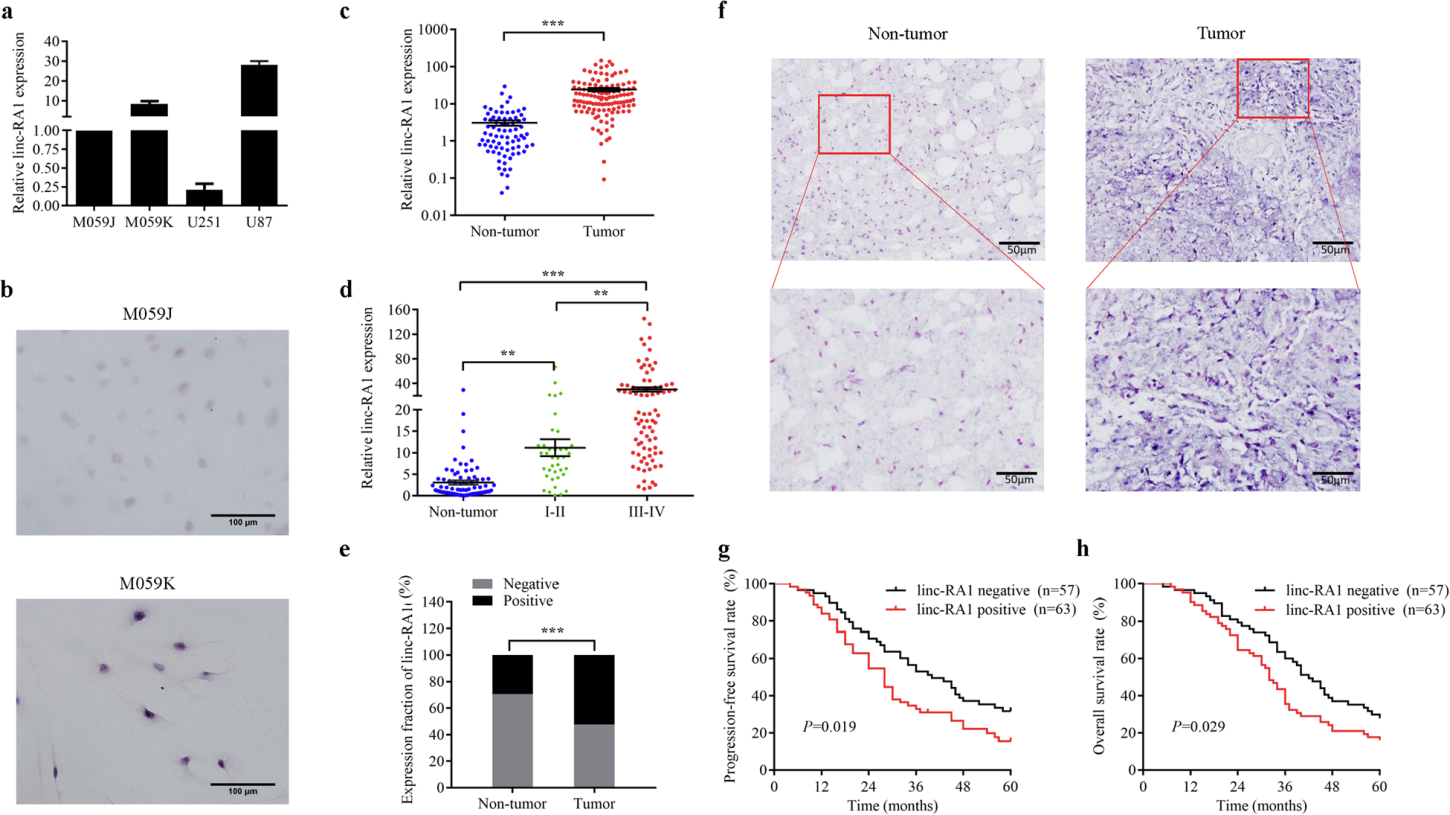
*Linc-RA1* is upregulated in glioma radioresistant cell lines and correlates with advanced glioma grades and poor prognosis. **a** *Linc-RA1* expression levels in glioma cell lines were detected using qRT-PCR. **b** Representative images of *linc-RA1* expression from M059J and M059K cells using an ISH assay. **c** *Linc-RA1* expression levels in nontumor brain tissues (*n* = 78) and glioma tissues (*n* = 120) were detected using qRT-PCR. **d** *Linc-RA1* expression levels in nontumor brain tissues (*n* = 78), low-grade (WHO I–II, *n* = 38), and high- grade (WHO III–IV, *n* = 82) glioma tissues were detected using qRT-PCR. **e** Expression fraction of *linc-RA1*, as detected using an ISH assay. **f** Representative images of *linc-RA1* expression from nontumor brain tissues and glioma tissues using an ISH assay. **g** Kaplan–Meier curves showing progression-free survival of 120 patients with glioma who were positive or negative for *linc-RA1* expression. **h** Kaplan–Meier curves showing overall survival of 120 patients with glioma who were positive or negative for *linc-RA1* expression.

To identify lncRNAs involved in radioresistance of glioma, the expression profile of lncRNAs between the M059J and M059K cells was assessed using microarray analysis in our previous study^16^. M059J and M059K cell lines are established from different areas of the same tumor^15^, with M059K cells that were much more resistant to IR than M059J cells (Supplementary Fig. 1a). Seventy-seven lncRNAs were expressed differently (fold change > 10.0, *p* value < 0.05), including 30 upregulated and 47 downregulated lncRNAs in M059K cells compared with that in M059J cells. Among them, lncRNA *TCONS_00009108* (GenBank Accession no. XR_949976.1), which was one of the top-scoring highly overexpressed lncRNAs in radioresistant cells, caught our attention and was named as radioresistance-associated long intergenic noncoding RNA 1 (*linc-RA1*). The full-length cDNA of *linc-RA1* was obtained using RACE (Supplementary Table 1). Subsequent analysis showed that *linc-RA1* was highly upregulated in radioresistant glioma cell lines (M059K and U87) compared with that in radiosensitive glioma cell lines (M059J and U251) (Fig. 1a). Consistently, high expression of *linc-RA1* was observed in M059K cells compared with that in M059J cells using ISH (Fig. 1b). Thus, the expression of *linc-RA1* in radioresistant glioma cells was significantly higher than that in radiosensitive glioma cells.

Furthermore, analysis of the expression of *linc-RA1* in 120 glioma tissues and 78 nontumor brain tissues revealed significantly higher expression in glioma tissues (Fig. 1c). Moreover, *linc-RA1* expression was upregulated in high-grade glioma tissues (WHO III–IV, *n* = 82) compared with that in low-grade tissues (WHO I–II, *n* = 38), indicating a positive correlation between *linc-RA1* expression and the malignancy degree of glioma (Fig. 1d). However, *linc-RA1* expression did not correlate with gender, age, or tumor size (Table 1). Consistently, the higher expression of *linc-RA1* in glioma tissues (52.50%, 63 of 120) compared with that in nontumor brain tissue samples (29.49%, 23 of 78, Fig. 1e, f and Table 2) was also confirmed using ISH. More importantly, Kaplan–Meier analysis revealed that patients with glioma with higher *linc-RA1* expression had significantly shorter progression-free and overall survival than those with lower expression (Fig. 1g, h). Thus, *linc-RA1* correlated with advanced glioma grades and poor prognosis of patients with glioma.

**Table 1:** Expression of *linc-RA1* and distribution of clinicopathologic factors in glioma patients.

Variable	Patients (*n*)	linc-RA1 expression	*P* value
*Gender*			
Male	71	26.825	0.223
Female	49	20.432	
*Age (y)*			
≤40	54	21.961	0.430
>40	66	26.059	
*Grade*			
Low (I+II)	38	15.977	0.028
High (III+IV)	83	28.033	
*Tumor size (cm)*			
≤4	31	20.858	0.443
>4	89	25.384

**Table 2:** Expression of linc-RA1 in nontumor tissues and glioma tissues.

	Nontumor	Glioma
No. of negative	55	53
No. of positive	23	67
Positive ratio (%)	29.49%	52.50%

### *Linc-RA1* enhances radioresistance of glioma cells in vitro and in vivo

**Fig. 2:**
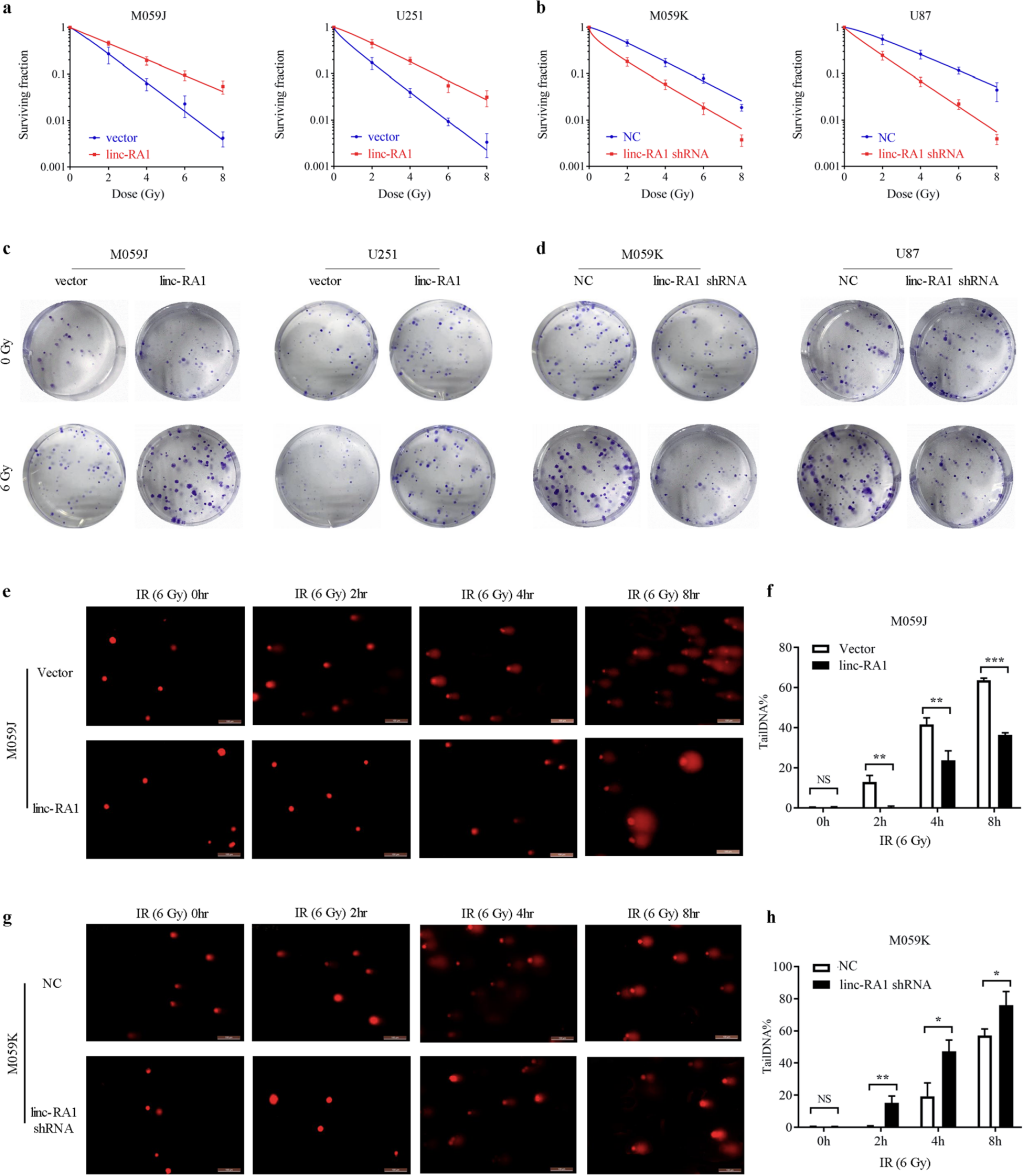
*Linc-RA1* enhances radioresistance of glioma cells in vitro. **a** Clonogenic survival assays of M059J and U251 cells transduced with vector or lentiviruses encoding human *linc-RA1* sequence. **b** Clonogenic survival assays of M059K and U87 cells transfected with scrambled shRNA or *linc-RA1* shRNA. **c** Representative images of clonogenic survival assays from M059J and U251 cells transduced with vector or *linc-RA1*. **d** Representative images of clonogenic survival assays from M059K and U87 cells transfected with scrambled shRNA or *linc-RA1* shRNA. **e**, **f** Representative images (**e**) and quantification (**f**) of comet assays of M059J cells transduced with vector or *linc-RA1*, at the indicated time points after 6-Gy IR. **g**, **h** Representative images (**g**) and quantification (**h**) of comet assays of M059K cells transfected with scrambled shRNA or *linc-RA1* shRNA, at the indicated time points after 6-Gy IR. Data are presented as means ± SD, *n* = 3, *P < 0.05, **P < 0.01, ***P < 0.001.

To evaluate the roles of *linc-RA1* in radioresistance of glioma cells, it was overexpressed in relatively radiosensitive M059J and U251 cells, and suppressed in relatively radioresistant M059K and U87 cells (Supplementary Fig. 1b, c). Overexpression or knockdown of *linc-RA1* had no significant influence on the viability of the glioma cells (Supplementary Fig. 1d, e). Interestingly, *linc-RA1* overexpression increased the surviving fractions of M059J and U251 cells (Fig. 2a, c), whereas suppression of *linc-RA1* decreased the surviving fraction of M059K and U87 cells (Fig. 2b, d). To measure DNA damage, we detected DNA breaks using comet assays, which showed that the tail DNA% (indicating DNA damage) was significantly lower in M059J cells overexpressing *linc-RA1* (Fig. 2e, f), and higher in M059K cells with downregulated *linc-RA1* expression (Fig. 2g, h), compared with control cells.

**Fig. 3:**
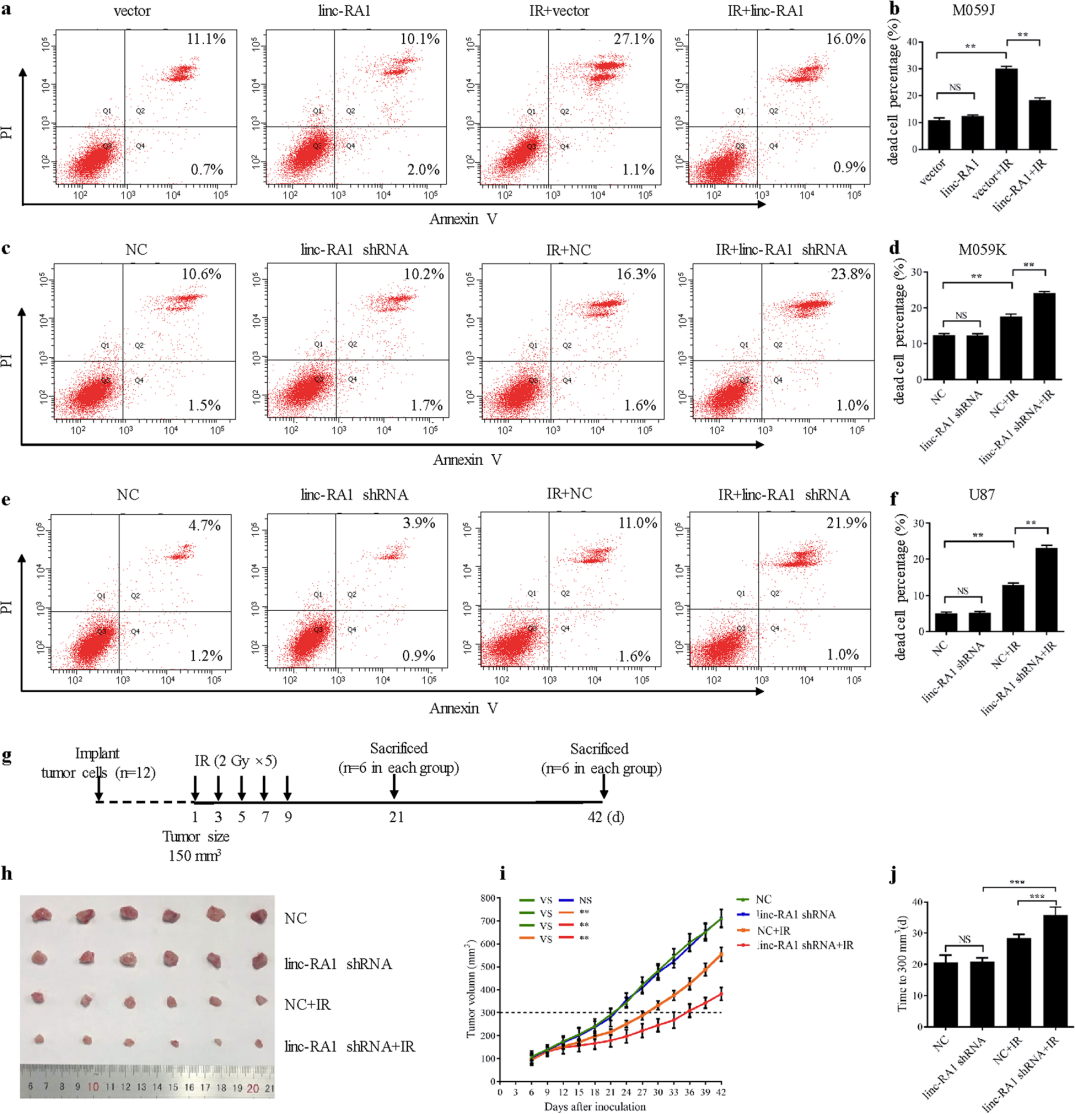
*Linc-RA1* enhances radioresistance of glioma cells in vitro and in vivo. **a**, **b** The percentage of irradiation-induced dead cells were measured with flow cytometry of M059J cells transfected with vector or *linc-RA1*, with or without the exposure to irradiation at a dose of 6 Gy. **c**, **d** The percentage of irradiation-induced dead cells were measured using flow cytometry of M059K transfected with scrambled shRNA or *linc-RA1* shRNA, with or without 6-Gy IR. **e**, **f** The percentage of irradiation-induced dead cells were measured using flow cytometry of U87 cells as indicated. Data are presented as means ± SD, *n* = 3, **P < 0.01. **g** Schemes for the establishment and treatment of the glioma mouse model. **h** Representative images of tumors in each group (*n* = 6) when the tumor volume in the control group (NC) reached approximately 300 mm^3^ on the 21st day after inoculation. **i** Growth curves of tumor volumes in each group (*n* = 6); bars, SE **P < 0.01. **j** Mean time for the tumor size to reach 300 mm^3^ in each group (*n* = 12); bars, SE ***P < 0.001.

Radiation damage can trigger IR-induced cell death by different pathways, such as mitotic catastrophe, necrosis, and apoptosis^17^. Our results showed that the combination of *linc-RA1* overexpression with IR significantly decreased the percentage of IR-induced dead cell (Fig. 3a, b). Similarly, the percentage of IR-induced dead cell was significantly higher when M059K and U87 cells with downregulated *linc-RA1* received IR (Fig. 3c–f).

To further verify these results, an in vivo tumor model was employed. U87 cells stably transduced with a scrambled shRNA or an shRNA targeting *linc-RA1* were injected subcutaneously into nude mice. When the tumor size reached approximately 150 mm^3^, xenograft tumors of the IR groups received local tumor IR with a fractionated dose of 2 Gy every other day, five times (Fig. 3g). The tumor size was measured until the tumors in all groups grew to 300 mm^3^ (Fig. 3h, i). In the control and *linc-RA1*-knockdown groups, the tumors reached 300 mm^3^ on days 21 and 22, respectively. Importantly, in the group treated with IR alone, the time to reach 300 mm^3^ was 27 days, and which increased to 36 days when IR was combined with knockdown of *linc-RA1* (Fig. 3j). The results showed that knockdown of *linc-RA1* alone had no significant influence on tumor growth, but did inhibit tumor growth after IR. Taken together, these results indicated that *linc-RA1* enhances the radioresistance of glioma cells in vitro and in vivo.

### *Linc-RA1* promotes the H2Bub1 modification after exposure to IR

**Fig. 4:**
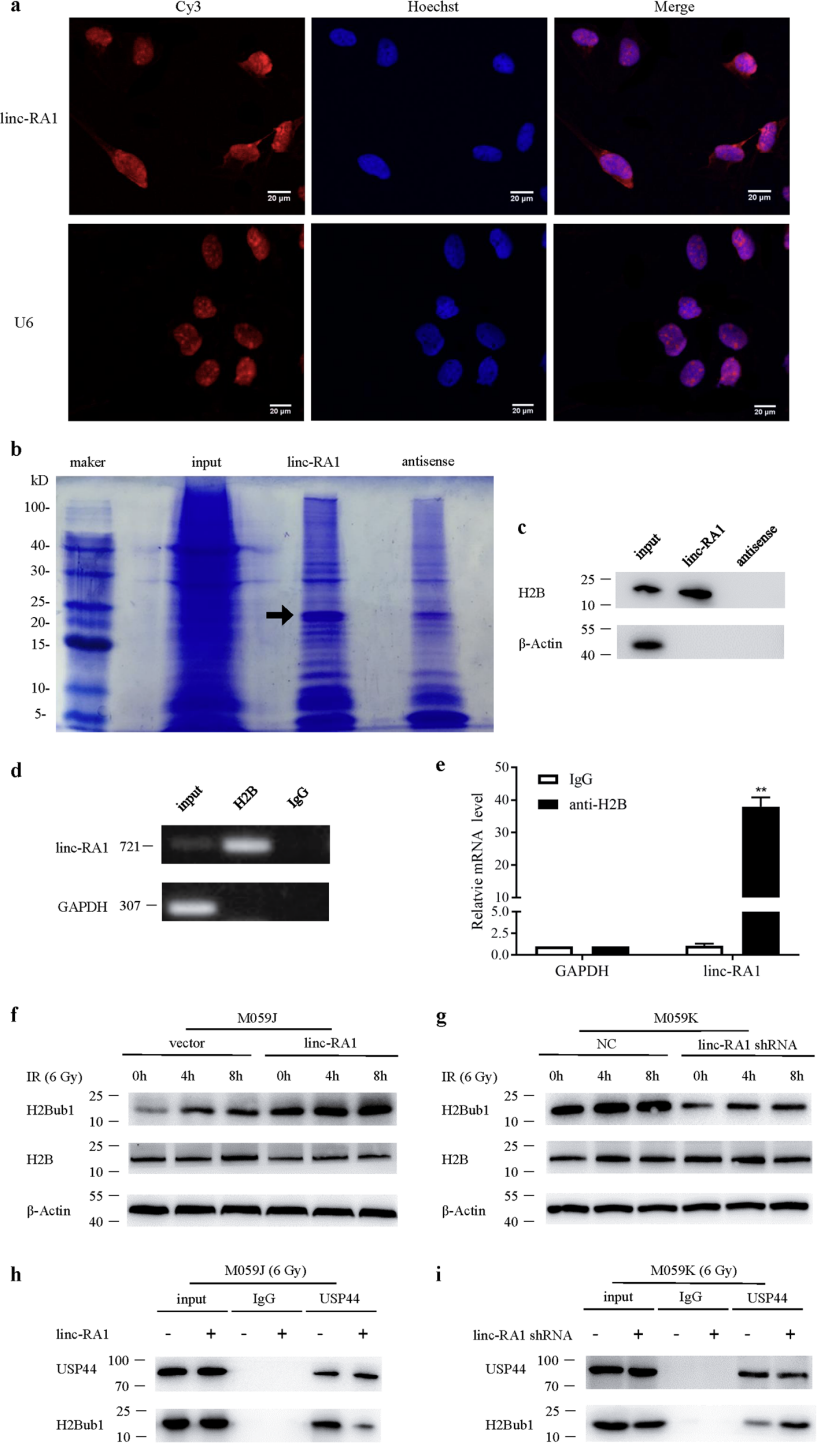
*Linc-RA1* promotes H2Bub1 modification with the exposure to irradiation. **a** FISH was used to detect the location of *linc-RA1*. **b** An RNA-pulldown assay was used to identify nuclear proteins that bind to *linc-RA1*, followed by mass spectrometry analysis. Highlighted regions (the arrow) were subjected to mass spectrometry for identification, and H2B was identified as the band unique to *linc-RA1*. **c** Western blotting analysis showing the specific interaction of *linc-RA1* with H2B. **d** RNA immunoprecipitation was performed using anti-H2B antibodies and specific primers to detect *linc-RA1* or GAPDH. **e** Enrichment of RNA immunoprecipitation was determined as the amount of RNA associated with immunoprecipitation of H2B relative to the input control. **f** Western blotting analysis of H2B and H2Bub1 levels in M059J cells transduced with vector or *linc-RA1*, at the indicated time points after 6-Gy IR. **g** Western blotting analysis of H2B and H2Bub1 levels in M059K cells transfected with scrambled shRNA or *linc-RA1* shRNA, at the indicated time points after 6-Gy IR. **h** M059J cells were transduced with vector or *linc-RA1*, followed by 6-Gy IR. Lysates were immunoprecipitated with anti-USP44 antibody, and the immunoprecipitates and input were analyzed by western blotting with the indicated antibodies. **i** M059K cells were transfected with scrambled shRNA or *linc-RA1* shRNA, followed by 6-Gy IR. Lysates were immunoprecipitated with anti-USP44 antibody, and the immunoprecipitates and input were analyzed by western blotting with the indicated antibodies.

The localization of lncRNAs can have important implications on their molecular functions and mechanisms. Thus, the distribution of *linc-RA1* was examined using FISH in M059K cells. We found that *linc-RA1* was localized predominantly in the nuclei, with some expression in the cytoplasm, indicating that *linc-RA1* might play a major role in the nuclei (Fig. 4a). Studies have shown that lncRNAs can function by interacting with proteins^18^. Thus, we identified nuclear proteins that could bind to *linc-RA1* using RNA pulldown, followed by mass spectrometry analysis. The results showed that *linc-RA1* could bind several proteins, including H2B, XPF, NUMB, and PCBP1. Among the proteins identified by mass spectrometry of the specific protein band for *linc-RA1*, H2B was also detected by western blotting (Fig. 4b, c). The RNA immunoprecipitation (RIP) experiment using anti-H2B antibodies in extracts from M059K cells showed enrichment of *linc-RA1* (but not GAPDH mRNA) versus a nonspecific IgG control (Fig. 4d, e). Histone H2B is distributed in the nucleus and is involved with the DNA-damage response and other important pathways; therefore, it was chosen for further mechanistic research.

To explore whether *linc-RA1* functions via binding to H2B to contribute to the radioresistance of glioma cells, we investigated whether the level of H2B was affected by *linc-RA1* after IR at a dose of 6 Gy. However, the results showed an unchanged level of H2B (Fig. 4f, g). Also, we detected the expression of XPF, NUMB, and PCBP1 with *linc-RA1* overexpression in M059J cells or *linc-RA1* knockdown in M059K cells. There was no change on their expression (Supplementary Fig. 2a–f). Interestingly, previous studies confirmed that some histone modifications of H2B are involved in the DNA-damage response^19^. Thus, we hypothesized that *linc-RA1* might be involved in the histone modification of H2B, which might be associated with the radioresistance of glioma cells. Among the histone modifications of H2B, H2B K120 monoubiquitination (H2Bub1) can promote DNA-damage response^20^. Thus, we suspected that *linc-RA1* might promote glioma radioresistance by regulating H2Bub1. Consistently, our results showed that *linc-RA1* altered the level of H2Bub1 after IR at a dose of 6 Gy. Overexpression of *linc-RA1* increased the level of the H2Bub1 modification in M059J cells after IR (Fig. 4f), while knockdown of *linc-RA1* decreased the H2Bub1 modification level in M059K cells (Fig. 4g).

Furthermore, the protein levels of related enzymes, including ubiquitin ligases of H2Bub1, RNF20, and RNF40, and the deubiquitinating enzyme of H2Bub1, USP44, were detected to investigate the molecular mechanisms of *linc-RA1*. The RNF20–RNF40 E3 ubiquitin ligase complex can monoubiquitylate histone H2B to produce H2Bub1, while the deubiquitinase USP44 can remove this modification^21,22^. The results showed that the levels of RNF20, RNF40, and USP44 were unchanged with *linc-RA1* upregulation or downregulation, indicating that *linc-RA1* regulates the monoubiquitination of histone H2B, independent of the expression level of ubiquitin ligases RNF20/40 or deubiquitinating enzyme USP44 (Supplementary Fig. 3a, b). Importantly, lncRNAs can also directly bind proteins that are essential for a signaling pathway, thus regulating protein–protein interactions and modulating their functions^18,23^. Thus, we speculated that *linc-RA1* might affect the interactions between H2Bub1 and RNF20, RNF40, or USP44. Interestingly, overexpression of *linc-RA1* decreased the interaction between H2Bub1 and USP44 in M059J cells after IR (Fig. 4h), while knockdown of *linc-RA1* increased the interaction between H2Bub1 and USP44 in M059K cells after IR (Fig. 4i). These results indicated that *linc-RA1* could stabilize the level of H2Bub1 by inhibiting the interaction between H2Bub1 and USP44.

### *Linc-RA1* regulates radioresistance through H2Bub1-mediated autophagy

**Fig. 5:**
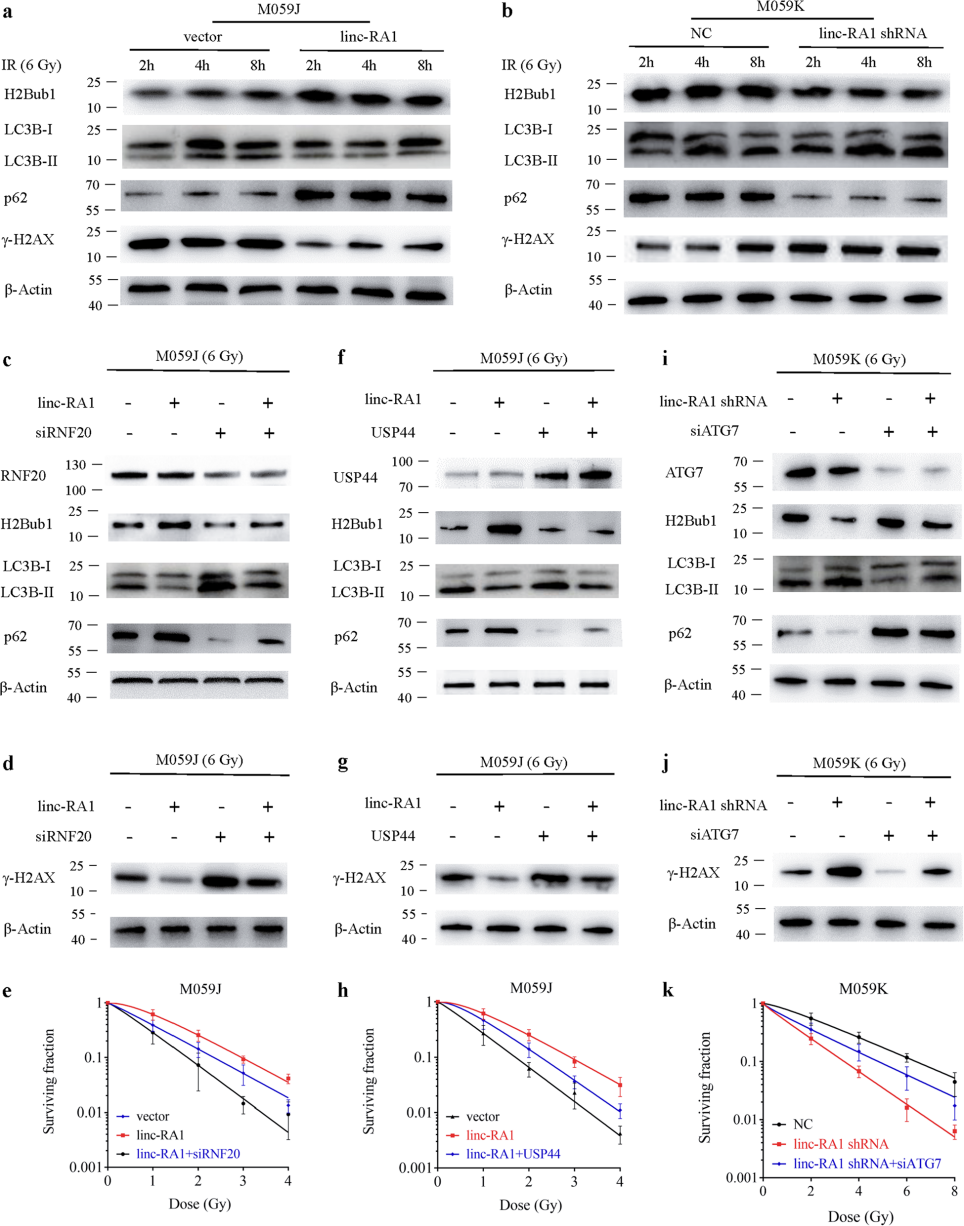
*Linc-RA1* regulates radioresistance through H2Bub1-mediated autophagy. **a** Western blotting analysis of H2Bub1, LC3B, p62, and γ-H2AX levels of M059J cells transduced with vector or *linc-RA1*, followed by 6-Gy IR. **b** Western blotting analysis of H2Bub1, LC3B, p62, and γ-H2AX levels of M059K cells transfected with scrambled shRNA or *linc-RA1* shRNA, followed by 6-Gy IR. **c** Western blotting analysis of H2Bub1, LC3B, and p62 levels of M059J cells that were transfected with vector or *linc-RA1*, and scrambled siRNA or RNF20 siRNA, followed by 6-Gy IR. **d** Western blotting analysis of γ-H2AX levels of M059J cells. M059J cells were treated as indicated. **e** Clonogenic survival assay of M059J cells. M059J cells were treated as indicated. **f** Western blotting analysis of H2Bub1, LC3B, and p62 levels of M059J cells that were transduced with vector or *linc-RA1*, and vector or USP44, followed by 6-Gy IR. **g** Western blotting analysis of γ-H2AX levels of M059J cells. M059J cells were treated as indicated. **h** Clonogenic survival assay of M059J. M059J cells were treated as indicated. **i** Western blotting analysis of H2Bub1, LC3B, and p62 levels of M059K cells that were transfected with scrambled shRNA or *linc-RA1* shRNA, and scrambled siRNA or ATG7 siRNA, followed by 6-Gy IR. **j** Western blotting analysis of γ-H2AX levels of M059K cells. M059K cells were treated as indicated. **k** Clonogenic survival assay of M059K cells. M059K cells were treated as indicated.

Several studies have indicated that the decrease in H2Bub1 levels induced by USP44 results in autophagy activation^24^. Importantly, autophagy is involved with DNA-damage response^25,26^. The induction of autophagy can enhance radiosensitivity^27,28^. Therefore, we hypothesized that *linc-RA1* could affect H2Bub1-mediated autophagy pathway, thereby promoting radioresistance of glioma cells. *Linc-RA1* overexpression in M059J cells at 6 h after exposure to 6 Gy led to an increase in H2Bub1 levels, accompanied simultaneously with a decrease in the microtubule-associated protein 1 light-chain 3 beta (LC3B)-II/I ratio and an increase in p62 (Sequestosome 1) levels. In contrast, suppression of *linc-RA1* in M059K cells at 6 h after exposure to 6 Gy reduced the level of H2Bub1 and p62, and increased the LC3B-II/I ratio (Fig. 5a, b). *Linc-RA1* overexpression decreased the level of γ-H2AX, which reflects the number of DNA double-stand breaks (DSBs), in M059J cells, while *linc-RA1* knockdown increased the level of γ-H2AX in M059K cells after IR (Fig. 5a, b). These results indicated that *linc-RA1* inhibited autophagy after IR in glioma cells.

We sought to determine whether *linc-RA1* promotes glioma radioresistance through H2Bub1. We found that H2Bub1 inhibition by RNF20 knockdown could partly restore the inhibition of autophagy caused by the overexpression of *linc-RA1* in M059J cells (Fig. 5c). H2Bub1 inhibition by RNF20 knockdown could partly restore the decrease of γ-H2AX level caused by the overexpression of *linc-RA1* in M059J cells (Fig. 5d). A clonogenic survival assay revealed that H2Bub1 inhibition by RNF20 knockdown could partly restore the enhanced radioresistance induced by *linc-RA1* overexpression in M059J cells (Fig. 5e). Consistently, H2Bub1 inhibition by USP44 overexpression also produced similar effects (Fig. 5f–h).

Furthermore, we also found that the knockdown of autophagy-related protein 7 (ATG7) partly restored the increased γ-H2AX level caused by knockdown of *linc-RA1* in M059K cells (Fig. 5i, j). A clonogenic survival assay revealed that ATG7 knockdown partly restored the enhanced radiosensitivity induced by *linc-RA1* knockdown in M059K cells (Fig. 5k). The treatment with autophagic inhibitor Spautin-1 also produced similar effects (Supplementary Fig. 4a–c).

**Fig. 6:**
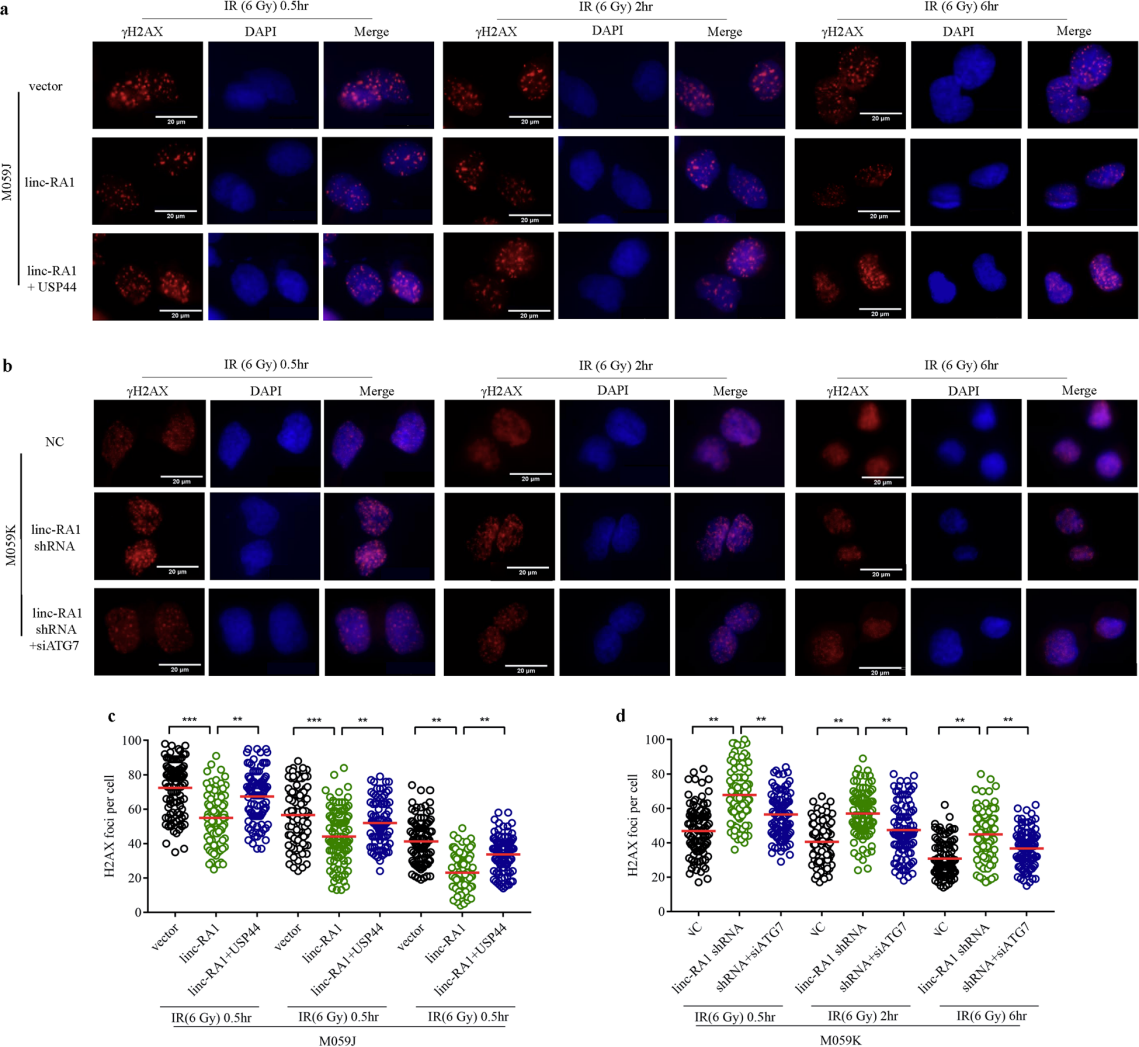
*Linc-RA1* regulates radioresistance through H2Bub1-mediated autophagy. **a** Representative immunofluorescence images of γ-H2AX foci of M059J cells at the indicated time points after 6-Gy IR. M059J cells were transduced with vector or *linc-RA1*, and vector or USP44. **b** Representative immunofluorescence images of γ-H2AX foci of M059K cells at the indicated time points after 6-Gy IR. M059K cells were transfected with scrambled shRNA or *linc-RA1* shRNA, and scrambled siRNA or ATG7 siRNA. **c** Quantification of γ-H2AX foci of M059J cells. M059J cells were treated as in (**a**). **d** Quantification of γ-H2AX foci of M059K cells. M059K cells were treated as in (**b**). Data are presented as means ± SD, *n* = 3, **P < 0.01, ***P < 0.001.

Moreover, immunofluorescence assays demonstrated that *linc-RA1* overexpression could decrease the foci number of γ-H2AX in M059J cells (Fig. 6a, c), while *linc-RA1* knockdown could increase the foci number of γ-H2AX in M059K cells (Fig. 6b, d). Moreover, H2Bub1 inhibition by USP44 overexpression could partly restore the decreased γ-H2AX foci number caused by *linc-RA1* overexpression in M059J cells (Fig. 6a, c). ATG7 knockdown could partly restore the increased γ-H2AX level caused by *linc-RA1* knockdown in M059K cells (Fig. 6b, d). Altogether, these results suggested that *linc-RA1* promoted radioresistance at least partly through the alteration of H2Bub1 level and the regulation of autophagy in glioma cells.

## Discussion

**Fig. 7:**
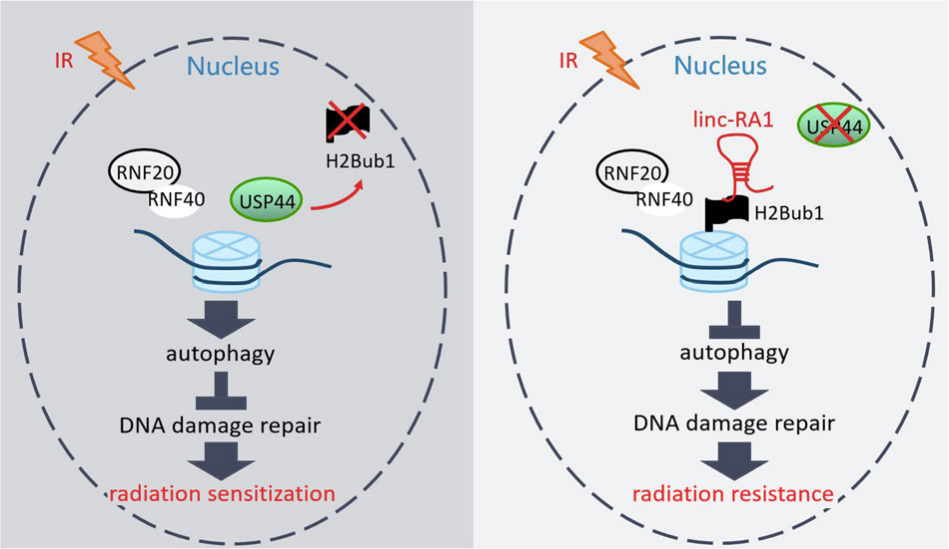
Schematic diagram for the working model of the regulation of radiosensitivity by linc-RA1. At low expression of linc-RA1, USP44 could catalyze H2Bub1 deubiquitination, thereby promoting autophagy activation and contributing to glioma cell radiosensitivity (Left). At high expression, linc-RA1 could stabilize H2Bub1 levels by inhibiting its binding with USP44, thereby inhibiting autophagy activation and contributing to glioma cell radioresistance (Right).

In the present study, lncRNA *linc-RA1* was identified as upregulated in radioresistant glioma cells and glioma tissues compared with radiosensitive cells and nontumor tissues. *Linc-RA1* promotes the radioresistance of glioma cells. Mechanistically, *linc-RA1* stabilizes H2Bub1 levels by inhibiting its binding with USP44, thereby inhibiting autophagy activation and contributing to glioma cell radioresistance (Fig. 7). These findings indicated that *linc-RA1* plays an important role in regulating the radiosensitivity of glioma cells.

Recently, several studies have indicated that lncRNAs are involved in cancer radioresistance^29,30^. In cervical cancer, lncRNA *LINC00958* regulates RRM2 by competing for miR‐5095, thereby regulating radiotherapy resistance^31^. In prostate cancer, lncRNA *UCA1* enhances tumor cell radioresistance by inhibiting cell-cycle progression^32^. However, the study is about the roles of lncRNAs in glioma radioresistance. In our previous study, we showed that lncRNA *SNHG18* promoted glioma radioresistance by inhibiting semaphorin5A. Here, we further analyzed glioma radioresistance-related lncRNAs. We demonstrated that ectopic expression of *linc-RA1* in M059J and U251 cells significantly enhanced radioresistance, whereas *linc-RA1* knockdown in M059K and U87 cells increased radiosensitivity. This is the first study to investigate the effect of *linc-RA1* on the radiation response of glioma cells. Tumor cell sensitivity to radiotherapy is one of the major influencing factors that determine the prognosis of patients with HGGs^33^. Thus, targeting the *linc-RA1* might be an effective method to enhance glioma radiosensitivity.

LncRNAs display characteristic tissue-specific and cell-type-specific expression patterns, which could be used as biomarkers to classify and prognose tumors^34^. Many researches indicate that the aberrant expression patterns of lncRNAs in clinical samples correlate with malignancy grade and histopathological differentiation, which are clinically important in the diagnosis and prognosis of glioma. For example, previous studies demonstrated that upregulated lncRNA *CRNDE* expression correlates with larger tumor size, higher WHO grade, and worse overall survival of patients with glioma^35^. In addition, the differential expression of *HOXA11-AS* in different subtypes of glioma suggested it as a biomarker to identify glioma molecular subtypes^36^. Similarly, we found that high expression of *linc-RA1* correlated with higher histopathological grade and poor prognosis of glioma. Thus, high expression of *linc-RA1* might be a potential biomarker to classify and prognose glioma. The identification of novel glioma biomarkers is important to study molecular mechanisms and improve prognosis.

Histones are small nuclear proteins that play key roles in DNA compaction. The histone tails of nucleosomes are the substrates for many post-translational modifications (PTMs), including acetylation, methylation, and ubiquitination^37^. Histones carrying these PTMs modulate the accessibility and compaction of chromatin, which regulates transcription, DNA-damage repair, and chromosome compaction^37^. Accordingly, histone PTM dysregulation contributes to oncogenesis, and the proteins essential for the addition and removal of certain PTMs are frequently altered in cancers^38–40^. The monoubiquitin moiety from lysine 120 of H2B (H2Bub1) is an important PTM of this core histone, and is involved in transcription, the DNA-damage response, and autophagy^41^. The H2Bub1 enzymatic cascade involves E3 RING finger ubiquitin ligases, generally accepted to be the RNF20–RNF40 complex, and deubiquitinases, including USP7, USP22, and USP44^19^. It has been suggested that the binding of lncRNAs could affect the availability of PTM sites or the binding between the PTM enzymes and their targets^42,43^. In this study, we demonstrated that *linc-RA1* increased H2Bub1 levels by preventing its binding to USP44, which catalyzes H2Bub1 deubiquitination. Thus, we hypothesized that the binding of *linc-RA1* to H2B would make it less accessible to USP44, which suggested a distinct mode of action of lncRNAs in histone modification.

Radioresistance is caused by various factors, including the intrinsic biology of tumor cells (with genetic and epigenetic alterations), and the extensive heterogeneity and tumor microenvironment of gliomas^33^. Autophagy is recognized as a double-edged sword in radioresistance, which seems to depend on tumor type, stage, genetic context, and the tumor microenvironment^44^. On the one hand, autophagy has cytoprotective effects in cancer treatment^45^. Some researchers believe that radiation-induced autophagy could represent a radioprotective mechanism in cancer cells^46^. On the other hand, considerable evidence shows that radiation alone, or in combination with different chemical agents, can activate autophagy, leading to increased cell death^45–47^. Moreover, many researchers consider that activating autophagy could lead to cell death, called autophagic cell death, contributing to radiosensitization in gliomas^27,28^. For example, GDC-0941, an autophagy- inducing agent, drastically increased the sensitivity of glioblastoma cells to the dual treatment (TMZ + IR)^48^. In our study, autophagy inhibition by knockdown of ATG7 or treatment with Spautin-1 rescued clonogenic capability of irradiated M059K cells with linc-RA1 knockdown, suggesting a pro-death autophagy contribution. Taken together, our results suggested that *linc-RA1* knockdown could enhance radiosensitivity by activating autophagy in glioma cells. Our study demonstrated a new working pattern of histone modifications that promoted glioma radioresistance.

Radiation damage can trigger IR-induced cell death by various processes, such as mitotic catastrophe, necrosis, and apoptosis^17^. Our results mainly showed that *linc-RA1* promoted radioresistance of glioma cells through autophagy. Our FACS data showed that the sum of Annexin-positive, PI-negative cell population (indicative of early apoptosis) and Annexin-positive, PI-positive cell population (indicative of late apoptosis) was significantly different, which included many ways of cell death, such as apoptosis and necrosis. We supposed that the sum represented the amount of IR-induced cell death, and its differences were generated by the expression of *linc-RA1*. Further research is needed to understand the deeper mechanisms.

In conclusion, *linc-RA1* was upregulated in radioresistant glioma cells, and its expression correlated with high histopathological grade and poor prognosis of glioma. Mechanistically, *linc-RA1* stabilizes H2Bub1 levels, thereby inhibiting autophagy activation, which contributes to glioma cell radioresistance. This study provides key insights into the roles and mechanisms of lncRNAs in glioma radioresistance, implicating *linc-RA1* as a biomarker and potential therapeutic target in glioma radioresistance.

## Materials and methods

### Patients and tumor samples

Tumor tissues were collected from 120 patients with glioma at Nanfang Hospital of Southern Medical University (Guangzhou, 510515, China) from January 2007 and January 2012. The study was approved by the hospital ethics committee, and all specimens were collected following written consent by the patients. A diagnosis of glioma was confirmed histopathologically.

### Cell culture

Human glioma cells M059J and M059K were obtained from the ATCC (Manassas, VA, USA) and cultured under conditions following the manufacturer’s instructions. U251 and U87 cells were from the Cell Bank of Type Culture Collection of the Chinese Academy of Sciences (Shanghai, China) and cultured following the manufacturer’s instructions. All the cells were cultured at 37 °C in a humidified incubator containing 5% CO_2_.

### 5′ and 3′ rapid amplification of cDNA ends (RACE)

Total RNA was isolated from M059K cells as described above. 5′-RACE was performed using a 5′-Full RACE Kit with TAP (Takara); 3′-RACE was performed using a 3′-Full RACE Core Set with PrimeScript RTase Kit (Takara) following the manufacturer’s instructions. The full-length sequence of *linc-RA1* is listed in Supplementary Table 1.

### Quantitative real-time reverse transcription PCR (qRT-PCR)

Total RNA was extracted using the TRIzol Reagent (Invitrogen, Waltham, MA, USA) and processed using DNase I (Takara, Dalian, China) according to the manufacturer’s instructions. After conversion to cDNA, quantitative polymerase chain reaction (qPCR) was carried out using a SYBR Green PCR kit (Takara). The data were normalized to the expression of *GAPDH* (encoding glyceraldehyde-3-phosphate dehydrogenase) as a reference gene. Primer sequences are listed in Supplementary Table 2.

### Western blotting

Cell were lysed using radioimmunoprecipitation assay (RIPA) buffer (Beyotime, Shanghai, China) with proteinase inhibitors (Beyotime) and phosphatase inhibitors (Beyotime). Proteins were then electrophoresed and blotted onto a membrane (Bio-Rad, Hercules, CA, USA). Nonspecific binding was blocked by incubating the membrane with 5% nonfat dry milk for 2 h. Membranes are incubated with primary antibodies overnight at 4 °C, followed by horseradish peroxidase-conjugated secondary antibodies. Finally, the immunoreactive proteins on the membranes were visualized using an ECL detection kit (Millipore, Billerica, MA, USA). Primary antibodies are listed in Supplementary Table 3.

### Lentiviral construction and transduction

The lentiviral vector expressing full-length human *linc-RA1* was constructed by Genechem (Shanghai, China) and used to transfect M059J and U251 cells to generate stable cells overexpressing *linc-RA1* that could be selected using puromycin. The hU6-sh-*linc-RA1*-Ubiquitin-EGFP-IRES-puromycin lentiviral vector (Genechem) expressing a short-hairpin RNS (shRNA) and that could be selected using puromycin, was used to knockdown *linc-RA1* expression in M059K and U87 cells.

### siRNA transfection

A small-interfering RNA (siRNA) targeting *RNF20* (encoding ring finger protein 20) was synthesized by RiBoBio (Guangzhou, China). Transfection was accomplished using the Lipofectamine 3000 reagent (Invitrogen) according to the manufacturer’s protocol.

### Plasmid construction and transfection

The USP44-overexpressing plasmids were obtained from Vigene Biosciences (Shandong, China). Transfection was accomplished using Lipofectamine 3000 reagent (Invitrogen) according to the manufacturer’s protocol.

### Clonogenic survival assay

Equal quantities of cells were seeded in plates in triplicate. The cells were then exposed to IR at the indicated doses (Varian2300EX, Varian, Palo Alto, CA). After incubation for 10–14 days, the cells were fixed and stained with 4% paraformaldehyde and 1% crystal violet, respectively. Colonies with more than 50 cells were counted using microscopy. Survival curves were generated using the multitarget single-hit model.

### Immunofluorescence assay

Cells were seeded and irradiated with a dose of 6 Gy after adhering. After 0.5, 2, or 6 h of IR, the cells were fixed and permeabilized using 4% paraformaldehyde and 0.1% Triton X-100 (Sigma, St. Louis, MO, USA). The cells were blocked with 1% goat serum and incubated with primary antibodies. The cells were then incubated with fluorochrome-conjugated secondary antibodies. Finally, the cells were then incubated with 2-(4-amidinophenyl)-1H-indole-6-carboxamidine (DAPI). H2A.X variant histone (γ-H2AX) foci were counted under a fluorescence microscope (Olympus BX63, Tokyo, Japan) for more than 100 cells in each group.

### Tumor radiosensitivity assay

Animal experiments were performed strictly according to the principles approved by the Committee on the Ethics of Animal Experiments of Guangzhou Medical University (Guangzhou, China). For the in vivo experiments, suspensions of 1 × 10^7^/0.2 ml *linc-RA1*-silenced or control U87 cells were subcutaneously inoculated into the right hind limb of 4-week-old female nude mice (*n* = 12 mice per group). When the tumor size reached about 150 mm^3^ (usually the 10th day), xenograft tumors of the IR groups received local tumor IR with a fractionated dose of 2 Gy every other day for 10 days. The mice were then sacrificed when the tumor volume in the control group (NC) reached approximately 300 mm^3^, which was usually the 21st day after inoculation (*n* = 6 mice per group). Tumor growth was measured until the tumor volume reached at least 300 mm^3^ on the 42nd day (*n* = 6 mice per group).

### In situ hybridization (ISH)

The expression of *linc-RA1* in clinical glioma specimens was detected using ISH, performed as previously described^49^. The sections were deparaffinized with xylene, rehydrated in serial dilutions of ethanol, and treated with 0.2 N HCL. After washing for 3 times, the sections were incubated in proteinase K (40 µg/mL, Promega) for 20 min and fixed with 4% paraformaldehyde for 10 min. The sections were reconstituted using hybridization solution and incubated at 56 °C overnight in a digoxigenin-labeled *linc-RA1* probe (Exiqon, Vedbaek, Denmark). After washing, the sections were blocked with 5% normal goat serum for 1 h at room temperature followed by incubation in an anti-digoxigenin alkaline phosphatase conjugate (Roche, Stockholm, Sweden) overnight at 4 °C. Colorimetric signals were obtained by incubating the sections in 5-bromo-4-chloro-3-indolyl phosphate (BCIP)/nitro-blue tetrazolium chloride (NBT) buffer in the dark for 4 h at room temperature. Nuclear fast red was used as the counterstain.

### RNA pulldown

RNA pulldown was performed as previously described^49^. Briefly, biotinylated *linc-RA1* was in vitro transcribed with the Biotin RNA Labeling Mix (Roche Diagnostics, Indianapolis, IN, USA) and T7 RNA polymerase (Roche Diagnostics), treated with RNase-free DNase I (Roche Diagnostics), and purified with the RNeasy Mini Kit (Qiagen). Nuclear protein from M059K cell extracts was then mixed with biotinylated RNA and incubated with streptavidin agarose beads (Invitrogen) at room temperature. The associated protein was detected by western blotting. Specific bands were excised and analyzed by mass spectrometry.

### RNA immunoprecipitation

RNA immunoprecipitation was performed as previously described^49^. Briefly, cells were lysed with RIPA buffer (Beyotime) containing proteinase inhibitors (Beyotime) and phosphatase inhibitors (Beyotime). Magnetic beads (Invitrogen) were preincubated with primary antibodies or anti-rabbit IgG (Cell Signaling Technology, Beverly, MA, USA) for 30 min. Then the lysates were immunoprecipitated with beads and rotated overnight at 4 °C. RNA was purified from RNA–protein complexes bounded to the beads and then was analyzed by qRT-PCR.

### Comet assay

The Comet assay (Trevigen, Gaithersburg, MD, USA) was performed on transfected cells at 0, 2, 4, and 8 h after IR (6 Gy), according to the manufacturer’s instructions.

### Immunoprecipitation

For the immunoprecipitation assay, we incubated cell lysates with anti-USP44 or normal rabbit IgG overnight at 4 °C. The mixture was then incubated with protein A/G magnetic beads (Bimake, Houston, USA) and rotated for 2 h at 4 °C. After four washes with lysis buffer, the proteins were separated using sodium dodecyl sulfate polyacrylamide gel electrophoresis.

### Statistical analysis

The data are presented as the mean ± SD from at least three independent experiments. Student’s *t* test and one-way analysis of variance were used to compare continuous variables, and the *χ*^2^ or Fisher’s exact tests were used to compare categorical variables. Survival curves were plotted using the Kaplan–Meier method and compared using the log-rank test. All statistical analyses were performed using SPSS 25 software (IBM Corp., Armonk, NY, USA). A *P* value < 0.05 was considered statistically significant.

## Supplementary information

Supplementary Figure 1

Supplementary Figure 2

Supplementary Figure 3

Supplementary Figure 4

Supplementary Figure 5

Supplementary Figure legends

Supplementary Table 1

Supplementary Table 2

Supplementary Table 3
