# Supplementary figures and images for: Linc-RA1 inhibits autophagy and promotes radioresistance by preventing H2Bub1/USP44 combination in glioma cells
Source: Cell Death Dis. 2020 Sep 15;11(9):758. doi: 10.1038/s41419-020-02977-x (PMC7492255; doi:10.1038/s41419-020-02977-x)

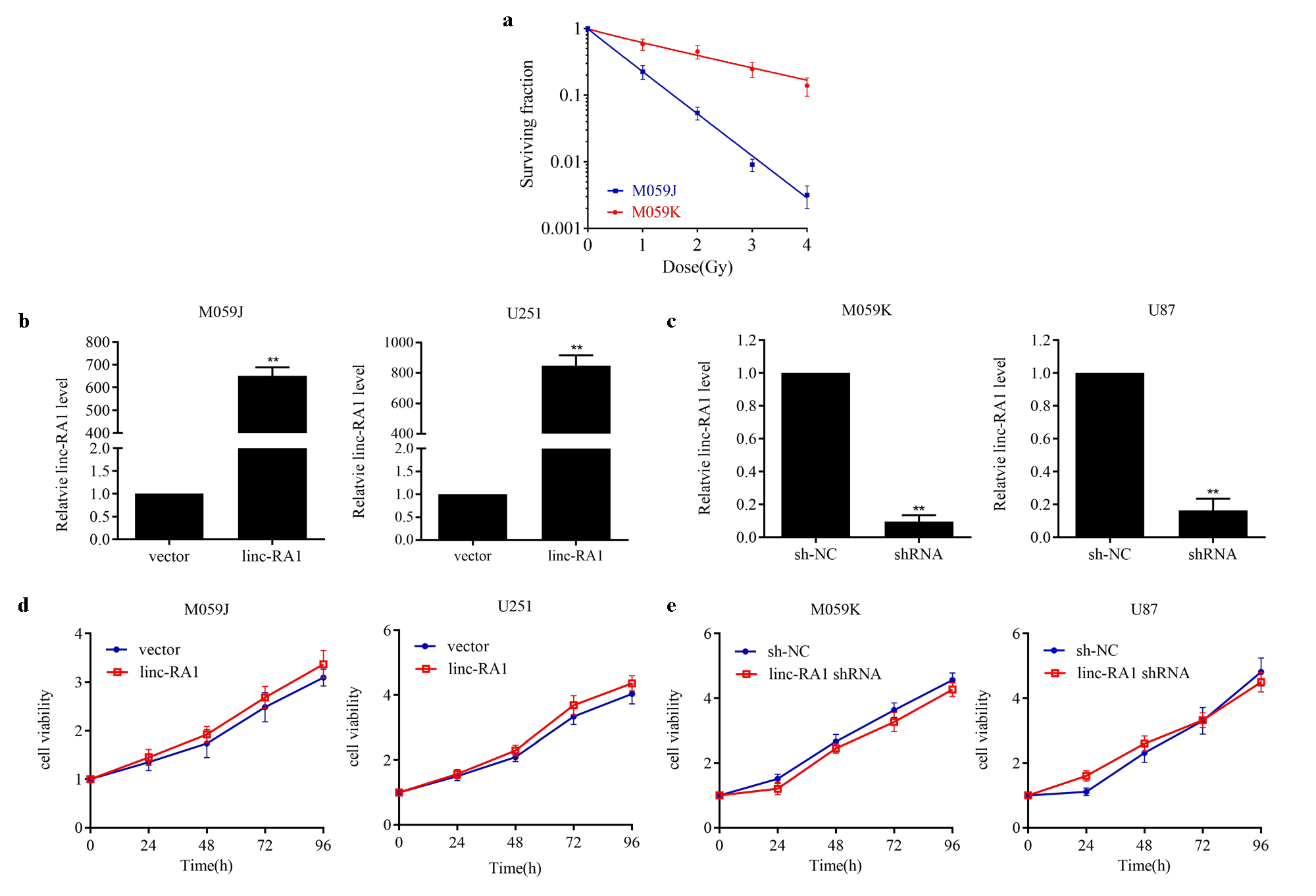

Supplement: Supplementary file 1 — Supplementary Figure 1 [file 41419_2020_2977_MOESM1_ESM.tif]

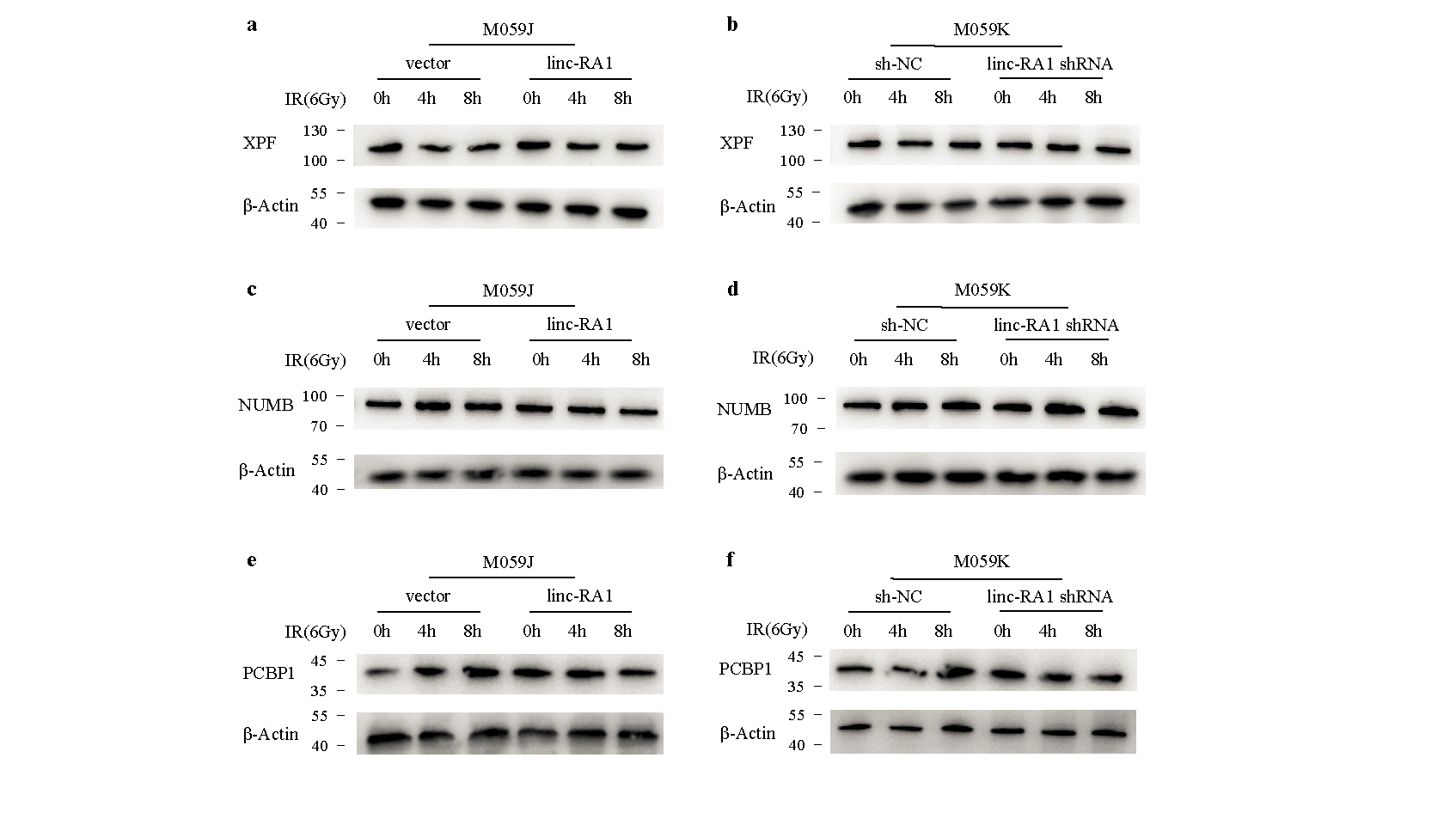

Supplement: Supplementary file 2 — Supplementary Figure 2 [file 41419_2020_2977_MOESM2_ESM.tif]

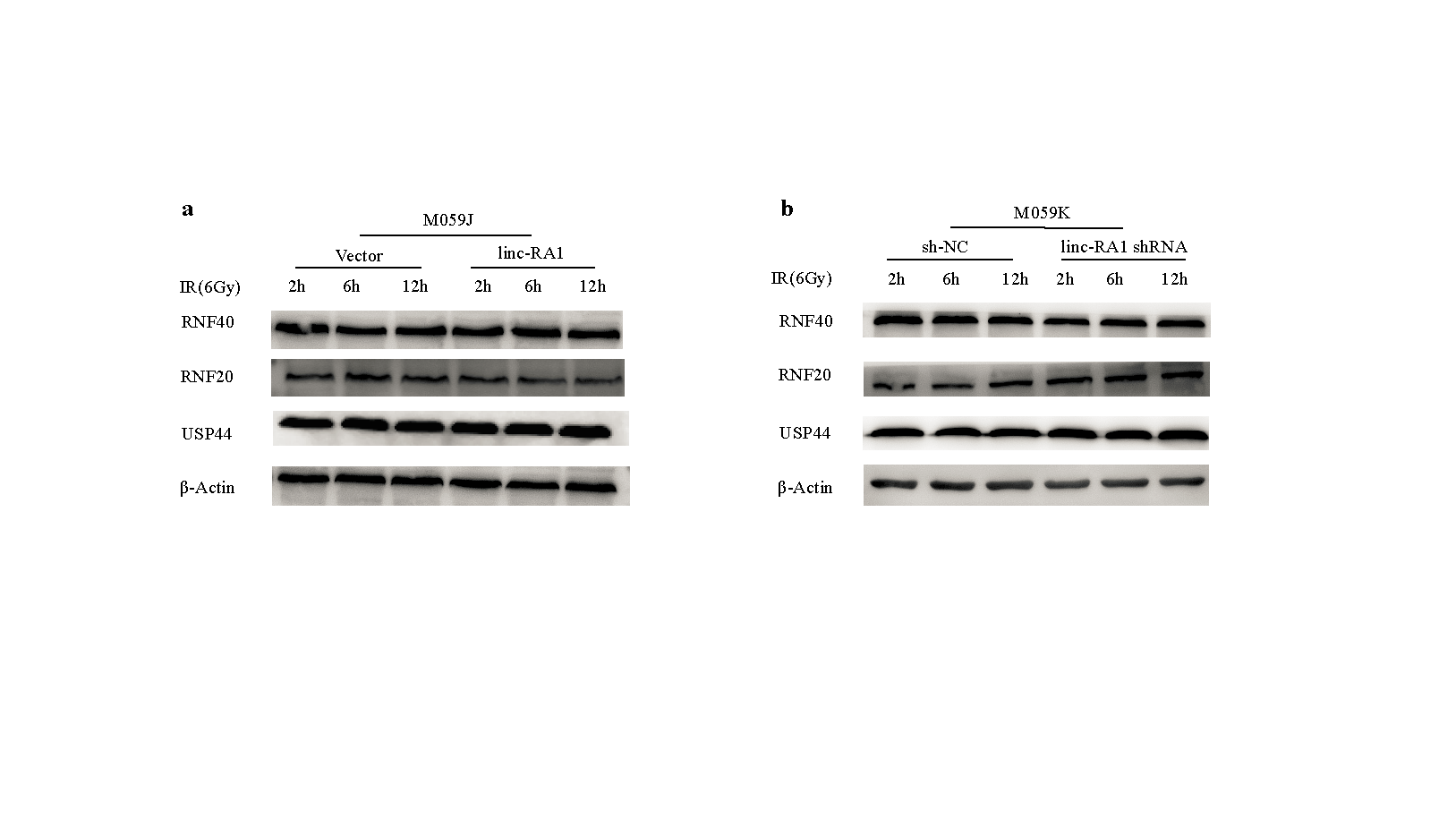

Supplement: Supplementary file 3 — Supplementary Figure 3 [file 41419_2020_2977_MOESM3_ESM.tif]

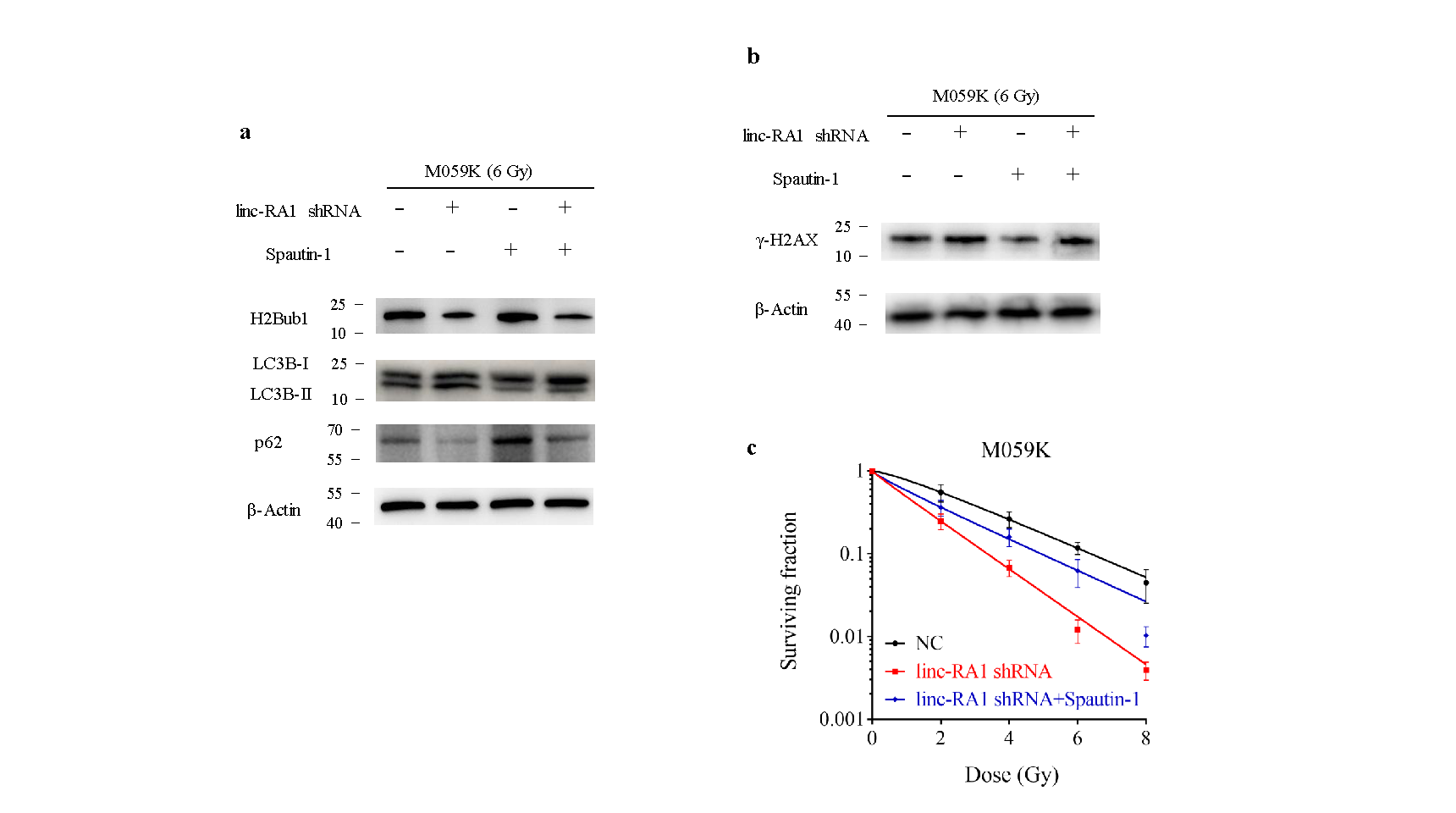

Supplement: Supplementary file 4 — Supplementary Figure 4 [file 41419_2020_2977_MOESM4_ESM.tif]

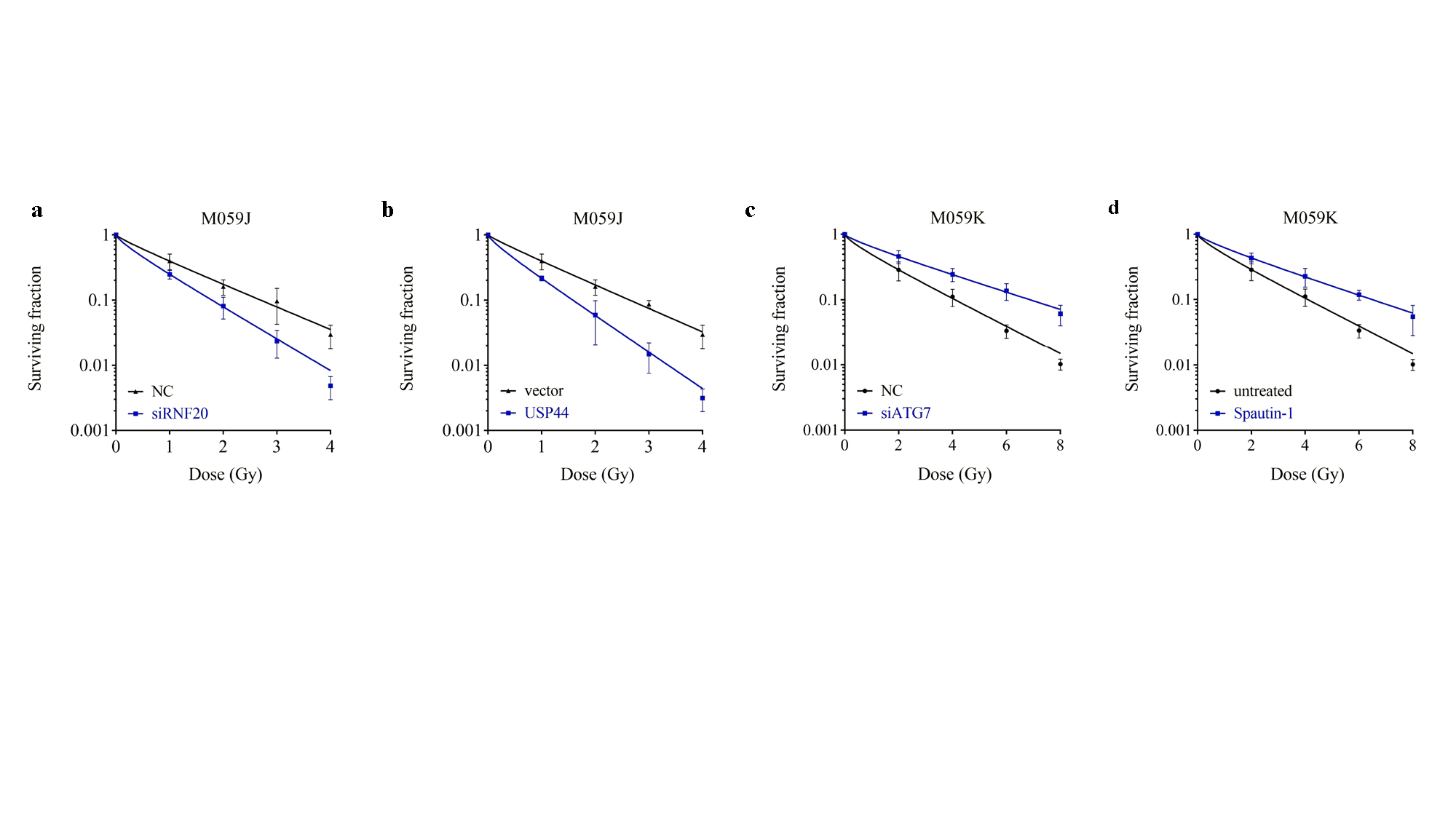

Supplement: Supplementary file 5 — Supplementary Figure 5 [file 41419_2020_2977_MOESM5_ESM.tif]
