# Supplementary material for: Linc-RA1 inhibits autophagy and promotes radioresistance by preventing H2Bub1/USP44 combination in glioma cells
Source: Cell Death Dis. 2020 Sep 15;11(9):758. doi: 10.1038/s41419-020-02977-x (PMC7492255; doi:10.1038/s41419-020-02977-x)
Supplement: Supplementary file 6 — Supplementary Figure legends [file 41419_2020_2977_MOESM6_ESM.docx]

**Supplementary Figure Legends**

**Supplementary Fig. 1. *Linc-RA1* had no significant influence on the viability of the glioma cells *in vitro.*** (a) Clonogenic survival assay of M059J and M059K cells. (b) *Linc-RA1* expression was detected by qRT-PCR in M059J and U251 cells transduced with vector or *linc-RA1*. (c) *Linc-RA1* expression was detected by qRT-PCR in M059K and U87 cells transfected with scrambled shRNA or *linc-RA1* shRNA. (d) Cell proliferation was detected by MTT assay in M059J and U251 cells transduced with vector or *linc-RA1*. (e) Cell proliferation was detected by MTT assay in M059K and U87 cells transfected with scrambled shRNA or *linc-RA1* shRNA.

**Supplementary Fig. 2.** ***Linc-RA1* had no significant influence on the expression of XPF, NUMB and PCBP1.** (a) Western blotting analysis of XPF levels in M059J cells transduced with vector or *linc-RA1*. (b) Western blotting analysis of XPF levels in M059K cells transfected with scrambled shRNA or *linc-RA1* shRNA. (c) Western blotting analysis of NUMB levels in M059J cells transduced with vector or *linc-RA1*. (d) Western blotting analysis of NUMB levels in M059K cells transfected with scrambled shRNA or *linc-RA1* shRNA. (e) Western blotting analysis of PCBP1 levels in M059J cells transduced with vector or *linc-RA1*. (f) Western blotting analysis of PCBP1 levels in M059K cells transfected with scrambled shRNA or *linc-RA1* shRNA.

**Supplementary Fig. 3. *Linc-RA1* regulates the monoubiquitination of H2B independent of the expression level of ubiquitin ligases RNF20/40 or deubiquitinating enzyme USP44.** (a) Western blotting analysis of RNF40, RNF20 and USP44 levels in M059J cells transduced with vector or *linc-RA1*. (b) Western blotting analysis of RNF40, RNF20 and USP44 levels in M059K cells transfected with scrambled shRNA or *linc-RA1* shRNA.

**Supplementary Fig. 4.** **The treatment with autophagic inhibitor Spautin-1 partly restored the enhanced radiosensitivity induced by *linc-RA1* knockdown in M059K cells.** (a) Western blotting analysis of H2Bub1, LC3B, and p62 levels of M059K cells which were transduced with scrambled shRNA or *linc-RA1* shRNA, with or without treatment of Spautin-1, followed by 6 Gy IR. (b) Western blotting analysis of γ-H2AX levels of M059K cells which were transfected with scrambled shRNA or *linc-RA1* shRNA, with or without treatment of Spautin-1, followed by 6 Gy IR. (c) Clonogenic survival assay of M059K cells which were transfected with scrambled shRNA or *linc-RA1* shRNA, with or without treatment of Spautin-1.

**Supplementary Fig. 5.** (a) Clonogenic survival assay of M059J cells which were transfected with scrambled siRNA or RNF20 siRNA. (b) Clonogenic survival assay of M059J cells which were transfected with vector or USP44. (c) Clonogenic survival assay of M059K cells which were transfected with scrambled siRNA or ATG7 siRNA. (d) Clonogenic survival assay of M059K cells with or without treatment of Spautin-1.
