# Supplementary material for: Linc-RA1 inhibits autophagy and promotes radioresistance by preventing H2Bub1/USP44 combination in glioma cells
Source: Cell Death Dis. 2020 Sep 15;11(9):758. doi: 10.1038/s41419-020-02977-x (PMC7492255; doi:10.1038/s41419-020-02977-x)
Supplement: Supplementary file 7 — Supplementary Table 1 [file 41419_2020_2977_MOESM7_ESM.docx]

**Table S1.** *Linc-RA1* sequence was detected by RACE.

*linc-RA1* sequence

5′-AGGAAGCAGATTTTCCCCCAGAGCATCCAGATAAGAACTCAGTCCACT CAACATCTTCAGCTTTGTGATATTCCAGGCATATTGTGCTGAACTTCTGAC TTACAGAACTACTGTACGCATTGGGCAAACTAGTTTCTGCGGTTAGCGCT GTGCCCACAAAGGCCTTGTCTCCACTCAAGCTAAAAAAAAAAAGAATCC AGAAAGCCTGGAAGATACTTTTCCAAGAATTTATTTTCTGTTTAGATTCTA CCCCTGAAAGTCAGTCCTGAAAGGATTATAAAAGCAAGAAAGAAACATG GACTCTAAGAATTAACAACAGTGTGGTACAGTCAGTCCTGAAAGGATTAT AAAAGCAAGAAAGAAACATGGACTCTAAGAATTAACAACAGTGTGGGA AGCCACGGAGGAGATTTATGACTACCCTAAATTGGTTTGTGTTTGGATGG ATGACTATTTCCTGCTCTTTATTACTAGAAATTGGCAGCAACATCTCAGAT TGGATAATGGTACTGGTGGAATAATTACCAGAAAGAAAAAAACATCCCCA CTAAAGTGATGAAAACATGTACAGATTGTCCAACCTTTTGCTTTTGTACC ACTTCCCCTTCCAGATGTCAGGGTTGTGATTTTGCTTTTGTACCACTTCCC CTTCCAGATGTCAGGGTTGTGATTTATTTCTCCCTAAGAGTTTAAAAATAA AACTCTCATCATAAAAAAAAAA-3′
