# Supplementary material for: Linc-RA1 inhibits autophagy and promotes radioresistance by preventing H2Bub1/USP44 combination in glioma cells
Source: Cell Death Dis. 2020 Sep 15;11(9):758. doi: 10.1038/s41419-020-02977-x (PMC7492255; doi:10.1038/s41419-020-02977-x)
Supplement: Supplementary file 8 — Supplementary Table 2 [file 41419_2020_2977_MOESM8_ESM.docx]

**Table S2.** Primer sequences used in qRT-PCR.

| Genes | Sequence |
| --- | --- |
| *linc-RA1* forward | 5′- GGCAGCAACATCTCAGATTGGA-3′ |
| *linc-RA1* reverse | 5′-CATATTTCCAGCCCAAAGT-3′ |
| GAPDH forward | 5′- GGCAGCAACATCTCAGATTGGA-3′ |
| GAPDH reverse | 5′- AGCCTTCTCCATGGTGGTGAAGAC-3′ |
