# Supplementary material for: Linc-RA1 inhibits autophagy and promotes radioresistance by preventing H2Bub1/USP44 combination in glioma cells
Source: Cell Death Dis. 2020 Sep 15;11(9):758. doi: 10.1038/s41419-020-02977-x (PMC7492255; doi:10.1038/s41419-020-02977-x)
Supplement: Supplementary file 9 — Supplementary Table 3 [file 41419_2020_2977_MOESM9_ESM.docx]

**Table S3.** Primary antibodies used in Western blotting.

| Antibody | Company | Catalog no. | Dilution |
| --- | --- | --- | --- |
| H2Bub1 | CST | 5546 | 1:1000 |
| H2B | abcam | ab52985 | 1:1000 |
| RNF20 | abcam | 32629 | 1:1000 |
| RNF40 | abcam | 191309 | 1:1000 |
| USP44 | Santa Cruz | sc-377203 | 1:200 |
| LC3B | CST | 3868 | 1:1000 |
| p62 | CST | 23214 | 1:1000 |
| γ-H2AX | CST | 9718 | 1:1000 |
| β-actin | CST | 8451 | 1:1000 |
